# A new, long-term root zone soil moisture dataset for operational agricultural drought monitoring over Africa

**DOI:** 10.1038/s41597-026-06585-w

**Published:** 2026-01-24

**Authors:** Ross I. Maidment, Tristan Quaife, Ewan Pinnington, Emily Black, Amsalework Ejigu, Dhirendra Kumar, Simon Thomas

**Affiliations:** 1 Department of Meteorology, University of Reading, Reading, UK; 2 National Centre for Earth Observation (NCEO), Reading, UK; 3 National Centre for Atmospheric Science (NCAS), Reading, UK; 4 European Centre for Medium Range Weather Forecasts (ECMWF), Reading, UK; 5 School of Agricultural, Policy and Development, University of Reading, Reading, UK; 6 School of Climate Change and Sustainability, Azim Premji University, Bengaluru, India; 7 European Satellite Services Provider (ESSP), Toulouse, France

**Keywords:** Natural hazards, Agriculture

## Abstract

Quantifying root zone soil moisture (RZSM) is critical for assessing water availability to crops and identifying agricultural drought across Africa, with access to reliable and timely RZSM data essential for informed decision-making. While rainfall is frequently used to assess crop growing conditions, it alone may not reliably reflect crop water availability due to the impact of evapotranspiration on soil moisture and rainfall not always reflective of concurrent soil water content at rooting depth. To provide robust information on agricultural drought, this paper describes a new, operational RZSM dataset, called TAMSAT soil moisture (TAMSAT-SM), available from 1983-present at 0.25° spatial resolution. TAMSAT-SM is derived using the JULES land surface model, forced with TAMSAT rainfall estimates and other meteorological variables from the NCEP reanalysis, and tuned to SMAP satellite soil moisture observations. Comparison against other RZSM products and independent satellite-derived vegetation health data show TAMSAT-SM can reliably capture the spatial and temporal RZSM and vegetation health patterns across Africa and can be a useful tool to support existing agricultural drought monitoring efforts.

## Background & Summary

Agricultural drought, which can be defined as the condition where there is insufficient soil moisture to meet the water demands of plants^1^, is often a result of prolonged precipitation deficit and is a recurrent peril across many parts of Africa that can lead to diminished crop yields – increasing food insecurity and impacting the livelihoods of communities it affects^2,3^. Recent notable examples include drought events affecting the Greater Horn of Africa (e.g. 2016–2017^4^; 2021–2022^5^ and Southern Africa in early 2024^6^. Common to all events is the need for humanitarian assistance to address the increased food insecurity. To support the mitigation of such events, it is important to be able to detect key agricultural drought characteristics such as onset, spatial extent and severity in near real-time (NRT). Moreover, to contextualise current events and gain a robust understanding of agricultural drought risk, it is crucial to have access to long-term, temporally consistent records of agricultural drought.

Currently, a wide range of variables are available to the user community for monitoring agricultural drought. Most frequently used is rainfall and derived indices, such as the Standardized Precipitation Index (SPI), which measures standardised anomalies in precipitation over a chosen time scale (e.g. 3 or 6 months). However, rainfall alone may not reliably reflect the water available to crops due to the impact of evapotranspiration on soil moisture – especially during periods of excess heat, and rainfall not being reflective of concurrent soil water content at rooting depth due to the antecedent properties of soil (SPI computed over a 3-month rolling window attempts to do this, yet may not accurately reflect actual soil water availability as it does not account for the impact of evaporative demand or plant transpiration). Other widely used variables include satellite-derived vegetation indicators such as the Normalized Difference Vegetation Index (NDVI), Vegetation Condition Index (VCI) and Enhanced Vegetation Index (EVI), all measures of vegetation greenness, and Vegetation Optical Depth (VOD) which measures vegetation water content as a proxy for biomass. In addition, combinations of variables related to atmospheric conditions affecting soil moisture can be used, such as the Standardized Precipitation Evapotranspiration Index (SPEI) which reflects climatic water balance anomalies over time by combining precipitation and potential evapotranspiration (PET). Moreover, satellite-derived land surface temperature (LST) and derived indices such as the Temperature Condition Index (TCI) can provide valuable information on drought by indicating surface thermal stress and temperature anomalies associated with vegetation and soil moisture deficits. Many existing datasets of the aforementioned variables provide over 20 years of records, with some exceeding 40 years, making them highly valuable for climate monitoring and analysing long-term drought variability and trends.

For agricultural drought however, the most relevant variable is soil moisture (in particular, low soil moisture during the crop growing season) as this is a direct measure of the water available to plants. Yet, this remains one of the most challenging land surface variables to observe over large areas. Despite their pin-point accuracy, *in-situ* measurements alone are far from adequate for routine agricultural drought monitoring with only a handful of stations providing routine and publicly accessible data^7^. Furthermore, satellite-derived soil moisture (e.g. such as those derived from Soil Moisture Active Passive (SMAP), Soil Moisture and Ocean Salinity (SMOS) and Advanced Scatterometer (ASCAT)) only provide an estimate of soil water content in the top 5–10 cm of soil, which is inadequate for inferring soil moisture available to the roots of crops that can exceed depths of 1 m when fully mature (hereinafter root zone soil moisture (RZSM)). As such, the only way to determine moisture availability at this depth is to model it, for example, by using a land surface model that can simulate the exchange of water and energy between the land surface and the atmosphere. While RZSM is similar to SPEI in that they both respond to precipitation deficits and evaporative demand, it can be argued that RZSM is better than SPEI for agricultural drought applications because it directly reflects soil water availability to plants, integrating both atmospheric conditions and land-surface processes, unlike SPEI, which is only based on the difference between precipitation and the atmospheric demand for water (PET) and not actual soil water availability.

Given the complexities of setting up a system to estimate RZSM combined with the technical challenges to implement operationally, there are a limited number of datasets that currently provide operational RZSM for Africa, and even fewer of those that provide long-term records exceeding 40 years (e.g. the Famine Early Warning Systems Network (FEWS NET) Land Data Assimilation System (FLDAS)). RZSM derived from satellite soil moisture observations also exist, such as those from the SMAP-L4 and SMOS-L4 products, but these also require the use of modelling to simulate soil moisture at depth and are shorter in length (i.e. SMOS from 2009 and SMAP from 2015), making these datasets less useful for assessing soil moisture anomalies and climate-related drought risk. A further sub-category of RZSM estimates can be obtained from model reanalyses such as the Fifth Generation of ECMWF Reanalysis (ERA5), the National Centers for Environmental Prediction (NCEP) and the Modern-Era Retrospective Analysis for Research and Applications (MERRA). These datasets benefit from consistent atmospheric forcings derived from model simulations combined with observations but can be subject to biases from poorly simulated precipitation^8,9^ and time-dependent biases from changes in the observing system^10^, thus reducing the skill and usability of such datasets.

Here, we describe and evaluate a new, operational and long-term (1983-present), daily soil moisture dataset (and related water balance variables) for Africa (Data Citation 1) produced by the Tropical Applications of Meteorology using SATellite and ground-based observations (TAMSAT) Group (hereinafter TAMSAT-SM), building on TAMSAT’s longstanding efforts to support drought monitoring across Africa since the early 1980s^11^. TAMSAT-SM has been derived using the Joint UK Land Environment Simulator (JULES) land surface model tuned to SMAP satellite soil moisture observations using data assimilation and forced with TAMSAT satellite rainfall estimates. We use JULES, a physically based land surface model, because it is easily configurable to run with user-specified meteorological forcings and offers extensive parameter and process customisation^12^. TAMSAT-SM currently provides soil moisture content for four soil depths (down to 3 m), a plant water stress variable called the soil moisture availability factor (commonly known as β (beta) amongst the JULES community) for five different plant types, and other water balance variables output by JULES (see Table 1 for details on the variables provided by TAMSAT-SM). Despite the availability of other RZSM products (see Table 4 for examples), to our knowledge, TAMSAT-SM is the first, operational soil moisture product for Africa that offers the combination of daily, long-term (+40 years) RZSM data updated in NRT (latency < 7 days) that is not dependent on reanalysis precipitation. Moreover, it is derived using a satellite rainfall product (TAMSAT) that is already established in many African users’ practices.

**Table 1:** Variables provided by the TAMSAT-SM v2.3.1 dataset.

Variable Name	Definition	Units
fsmc	PFT (plant function type) soil moisture availability factor (beta). Available for the five plant types: broadleaf trees, needleleaf trees, C3 grasses, C4 grasses, and shrubs. For agricultural drought applications, fsmc for C4 grasses is recommended as this is representative of major staple crops grown across sub-Saharan Africa, including maize, sorghum and millet, which share similar C4 physiology and soil moisture response characteristics (i.e. high water use efficiency under high temperature and light conditions). This variable (beta) relates how much plant growth is restricted by the available soil moisture; range 0–100 where 0 = plant has wilted and will not recover; 100 = plant growth not constrained by soil moisture.	No units
fsmc_gb	Gridbox soil moisture availability factor (beta)	No units
smcl	Moisture content of each soil layer for depths of 0–10 cm, 10–35 cm, 35–100 cm and 100-300 cm	kg m^−2^
smc_avail_top	Gridbox available moisture in the top 100 cm of soil	kg m^−2^
precip	Gridbox precipitation rate (TAMSAT v3.1 daily rainfall)	kg m^−2^ s^−1^
fqw_gb	Gridbox moisture flux from surface (actual evapotranspiration)	kg m^−2^ s^−1^
fao_et0	FAO Penman-Monteith evapotranspiration for reference crop (potential evapotranspiration)	kg m^−2^ s^−1^
runoff	Gridbox runoff rate	kg m^−2^ s^−1^

A principal strength of the TAMSAT-SM product is that all variables provided are consistent with one another. This offers two significant advantages: (1) the soil moisture outputs (*fsmc, fsmc_gb, smcl, smc_avail_top*) maintain a consistent mass balance with TAMSAT rainfall estimates, which have previously demonstrated high skill across Africa^13–17^. This consistency is critical, as precipitation deficits are the primary driver of agricultural drought in many regions of Africa^18,19^; (2) the water-balance variables provided (*precipitation, fqw_gb, fao_et0, runoff*) are internally consistent, ensuring that the water budget is largely balanced. This consistency is crucial for water budget studies across Africa and for applications requiring accurate estimates of actual or potential evapotranspiration. A further advantage of the TAMSAT-SM soil moisture estimates is their compatibility with the TAMSAT-AgriculturaL Early waRning sysTem (TAMSAT-ALERT) soil moisture forecasts^20–23^, which provide projections out to 160 days. This allows users to seamlessly combine NRT TAMSAT-SM soil moisture estimates with the TAMSAT-ALERT forecasts - an ability that, to our knowledge, is currently not available to African users by any other operational soil moisture estimation system.

Moreover, users often face challenges using soil moisture data due to issues such as limited access, complex data formats, and the need for expert knowledge to interpret the products correctly. However, there is a growing demand for soil moisture information across sectors such as agriculture, drought management, and index insurance, and part of TAMSAT-SM’s novelty lies in making such data easier to access and use, lowering barriers for non-specialist users - especially as the soil moisture is served in the same way as the widely used TAMSAT rainfall estimates, which many potential users of the soil moisture estimates are already familiar with.

In January 2025, the TAMSAT Group released version 2.3.1 of the soil moisture dataset. Given the paucity of *in-situ* soil moisture observations across Africa, the evaluation of TAMSAT-SM v2.3.1 presented in this paper has been split into two parts; (1) comparison of RZSM against existing RZSM datasets commonly used in agricultural drought applications and (2) benchmarking the soil moisture availability factor against independent satellite-derived vegetation health data. The evaluations have been carried out on a continental scale (for the RZSM comparisons) and at the regional scale (for the soil moisture availability factor comparisons).

## Methods

### Soil moisture estimation using JULES

The TAMSAT-SM product is produced using the community-led JULES (version 5.3) land surface model which has evolved from the UK’s Met Office Surface Exchange Scheme (MOSES) and is a core component of the UK Earth System Model (UKESM). It is a tool to study land surface processes by simulating interactions between the Earth’s land surface and the atmosphere by representing key physical processes such as energy exchange, water cycling, and carbon fluxes, including soil moisture dynamics, runoff, and evapotranspiration. While a full description of the energy and water fluxes within JULES can be found in Best *et al*.^24^, and the carbon fluxes and vegetation dynamics in Clark *et al*.^25^, here we provide an overview of the estimation of soil moisture within JULES.

In JULES, soil moisture is estimated through the representation of the vertical movement and retention of water within soil layers, by balancing inputs (precipitation) with outputs (evaporation, transpiration, and runoff). In the default configuration of JULES used in the generation of TAMSAT-SM, the soil is divided into multiple layers (0–10 cm, 10–35 cm, 35–10 cm and 100–300 cm – representing thicknesses of 10 cm, 25 cm, 65 cm and 200 cm respectively). Water enters the soil surface via infiltration at the top of the soil moisture column, while below the surface, water moves vertically between soil layers based on hydraulic conductivity and moisture gradients, calculated using the Richards equation and Darcy’s law. Soil hydraulic properties, which influence how water moves and is retained within each layer, are defined using a set of pedotransfer functions that relate the soil texture (i.e. the fractions of sand, silt, and clay) to parameters that include the saturated hydraulic conductivity and the wilting and critical points. Moisture is removed from the soil through either evaporation from the top soil layer or through transpiration via uptake by roots in the sub-surface layers. Runoff generated by JULES includes both the surface runoff, which occurs when precipitation exceeds surface and canopy evaporation, and subsurface runoff, which arises from water that exits the bottom most soil layer when deeper soil layers are saturated.

JULES also estimates the parameter β, which is used to represent soil moisture availability to plants (i.e. plant water stress). It is a dimensionless factor that compares the soil moisture content over the prescribed rooting depth to the amount of soil moisture needed for optimal plant growth (i.e. no plant water stress) and ranges between 0 and 1. The soil moisture availability factor β is calculated as follows:

1 $$\beta =\{\begin{array}{cc}1 & \theta \ge {\theta }_{c}\\ (\theta -{\theta }_{w})/({\theta }_{c}-{\theta }_{w}) & {\theta }_{w}\, < \,\theta \, < \,{\theta }_{c}\\ 0 & \theta \le {\theta }_{w}\end{array}$$

Where θ is the soil moisture content of the root zone (a weighted average determined by the plant functional type (PFT) rooting depth – see Table 2), θ_c_ is the critical point – the soil moisture concentration below which plants show water stress, and θ_w_ is the wilting point – the soil moisture concentration below which plants cannot extract water. When soil moisture is at or above θ_c_ (β = 1), the plant is not constrained by soil moisture and optimal growth occurs. If soil moisture is at or below θ_w_ (β = 0), water stress is severe enough that the plant wilts and dies. If soil moisture is between θ_w_ and θ_c_ (0< β < 1), plants will grow, but growth is constrained by a soil moisture deficit. For the TAMSAT soil moisture product, β is expressed as a percentage – i.e. with values ranging between 0–100.

**Table 2:** Rooting depths for JULES plant functional types (PFTs), indicating the maximum depth of soil moisture uptake.

PFT	Description	Rooting depth (m)
Broadleaf trees	Deciduous broadleaf trees	3.0 m
Needleleaf trees	Evergreen needleleaf trees	3.0 m
C3 grass	Temperate (cool-season) grass	1.0 m
C4 grass	Tropical (warm-season) grass (e.g., maize, sorghum, savanna grass)	1.0 m
Shrubs	Short woody plants	2.0 m

JULES represents multiple land surface types, including vegetation, soil, and urban areas, with vegetation categorised into five Plant Functional Types (PFTs); broadleaf trees, needleleaf trees, shrubs, C3 grasses, and C4 grasses.

### Meteorological forcing data

The JULES model has been forced with precipitation from TAMSAT (v3.1) and other atmospheric variables from the National Center for Environmental Prediction/National Center for Atmospheric Research (NCEP/NCAR) Reanalysis 1 (hereinafter NCEP) (see Table 3 for all variables required by JULES). The TAMSAT rainfall estimates, described in^14,15,26^, are based on Meteosat satellite thermal infra-red imagery which is used to identify deep, precipitating convective storm clouds by deriving maps of cold cloud duration (CCD) – the length of time a cloud top is colder than a given temperature threshold. Maps of CCD are calibrated using historical rain gauge information to provide Africa-wide rainfall estimates from 1^st^ January 1983 to present. Data are available on a 0.0375° × 0.0375° grid (~4 km) at daily, pentadal, dekadal, monthly and seasonal time-steps, with a latency of 2 days. The TAMSAT rainfall estimation approach has been assessed in multiple regions across Africa, demonstrating that TAMSAT rainfall estimates have comparable or better skill than many of the other widely used, long-term satellite rainfall datasets available for Africa^13–17^. The longevity (+40 years) and temporally stability of the TAMSAT rainfall record, availability of daily rainfall information and proven skill across most regions of Africa make it well-suited for the generation of long-term soil moisture time-series using JULES. The temporally complete version of the TAMSAT v3.1 dataset (variable: *rfe_filled*) has been used to generate TAMSAT-SM to provide a daily soil moisture time-series with no missing days.

**Table 3:** Meteorological forcing data required by JULES.

Variable	Units
Precipitation	Kg m^−2^ s^−1^
Air temperature at 2 m (daily mean, minimum and maximum)	K
Land surface (skin) temperature	K
Downward longwave radiation at the surface	W m^−2^
Downward shortwave radiation at the surface	W m^−2^
*U-* and *V-* components of wind	m s^−1^
Specific humidity at 2 m	Kg kg^−1^
Surface pressure	Pa

The NCEP reanalysis^27^ is a global atmospheric reanalysis dataset that combines historical weather observations with a consistent numerical model using data assimilation techniques, providing a continuous record of atmospheric variables from 1948 to present. Data are available on a 2.5° × 2.5° grid at multiple time-steps (6-hourly, daily and monthly). We currently use NCEP data to drive the JULES simulations, primarily because it provides freely available, continuous data with NRT availability (2–3 day latency), making it well-suited for operational applications. However, we acknowledge that the European Centre for Medium-Range Weather Forecasts (ECMWF) ERA5 offers significant advantages over NCEP, including higher spatial resolution and are actively considering transitioning to ERA5 forcing for future versions of TAMSAT-SM since the availability of ERA5T in NRT. To generate TAMSAT-SM, we have used daily values of both TAMSAT rainfall and the NCEP meteorological forcing data from 1^st^ January 1983 to the present, with all input datasets converted to a spatial resolution of 0.25° × 0.25° - the same resolution of the JULES outputs (see Table 1).

### Tuning of JULES soil hydraulic properties using SMAP

Soil hydraulic properties are supplied to JULES as ancillary data fields that vary spatially. They parameterise equations that govern, for example, the rate of flow of water through the soil and the holding capacity at saturation. The values of the soil hydraulic parameters are determined from data on soil bulk texture (e.g. percentage sand, silt and clay) by applying a set of pedotransfer functions. For the JULES runs described in this paper, the SoilGrids dataset^28^ has been used as an input to generate the soil hydraulic parameters. To tune the JULES soil moisture to SMAP (L3) observations we use a data assimilation technique called 4DEnVar to adjust the parameters of the pedotransfer functions to bring the modelled soil moisture in the top layer (10 cm) closer to the SMAP data. This procedure closely follows that applied in the UK by Pinnington *et al*.^12^ although the spatial resolution of the model grid used here is 0.25° × 0.25° and we assign the nearest SMAP observation to each grid cell. The data assimilation is carried out for a single year (2017), and we tested that the hindcast skill was approximately equal to that over the assimilation window (results not shown), indicating that improvement in the modelled soil moisture with the new soil hydraulic properties would persist in time.

An advantage of taking the approach of tuning the soil hydraulic parameters, as opposed to a more traditional data assimilation in which the model state is adjusted each time an observation becomes available, is that the adjustments we make to JULES persist in time. In effect, it is a mechanism for accounting the bias between JULES soil moisture estimates and observations from the SMAP mission. In addition, because the pedotransfer functions are assumed to be applicable globally in normal JULES operation, it means that our procedure adjusts the soil hydraulics for the entire land surface of the African continent.

### Product generation

**Fig. 1:**
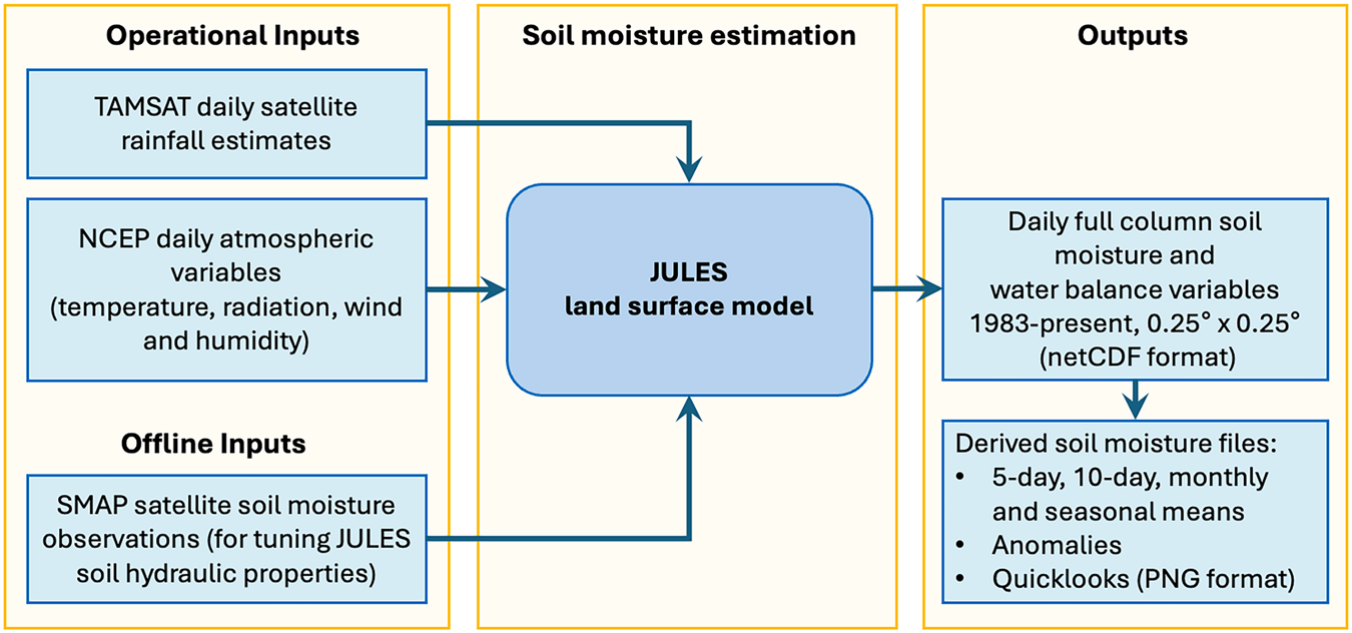
Overview of the TAMSAT soil moisture estimation system.

The generation of TAMSAT-SM is carried out within the UK’s Natural Environment Research Council (NERC)-funded high-performance computing JASMIN facility designed to support climate, weather, and environmental data analysis and processing, and is summarised in Fig. 1. Processing is managed by a Rose-Cylc workflow, which automates the execution of the JULES TAMSAT-SM suite when new meteorological forcing data are available. When new forcing data become available, JULES is automatically run from the end date of the previous cycle to the end date of the newly available driving data. Newly created JULES files are then post-processed before being archived within the Centre for Environmental Data Analysis (CEDA) repository (https://catalogue.ceda.ac.uk/uuid/083f0a37e058495eaef542263198019f/) and also made available through the TAMSAT website (https://www.tamsat.org.uk). Derived soil moisture files (e.g. temporally aggregated data and anomalies, as well as quicklooks in PNG format) are also created and accessible from the TAMSAT website.

## Data Records

### Data archive

The TAMSAT-SM dataset comprises of daily values of soil moisture (and related variables) from 1^st^ January 1983 to the delayed present on a 0.25° longitude by 0.25° latitude grid with data available for the African continent, Madagascar and parts of the Arabian Peninsula (domain coverage is 17.875°W to 51.375°E, 35.375°S to 37.375°N). This resolution was chosen as it offers a practical compromise between the differing spatial resolutions of the meteorological forcing datasets (NCEP (2.5°) and TAMSAT rainfall (0.0375°)), while remaining computationally feasible for NRT processing. Given that there are no gaps in either the TAMSAT v3.1 rainfall record (missing days in v3.0, as described in Maidment *et al*.^15^, have since been filled in v3.1 using a quantile-matching approach with the equivalent CHIRPSv2.0 rainfall estimates) or the NCEP reanalysis, the TAMSAT-SM dataset is temporally complete.

### Data access, format and file structure

The TAMSAT-SM dataset is provided in netCDF format and are freely available from the CEDA archive (https://catalogue.ceda.ac.uk/uuid/083f0a37e058495eaef542263198019f/) under a Creative Commons Attribution 4.0 International license (CC BY 4.0). The soil moisture and related variables are provided as one file per day. All variables are also available from the TAMSAT website (https://www.tamsat.org.uk) while the variable soil moisture availability factor for C4 plants (β) can be spatially and temporally subset using TAMSAT’s data subsetting service (accessible from the TAMSAT website). This subsetting service also provides data temporally aggregated to pentadal (5-day), dekadal (10-day), monthly and seasonal values, consistent with the TAMSATv3.1 rainfall estimates – thus allowing existing users of TAMSAT rainfall estimates to easily integrate TAMSAT-SM data into their operational or research applications. Users can also access the TAMSAT-ALERT soil moisture forecasts from the subsetting service, which provides soil moisture forecasts out to 160 days that are consistent with the historical soil moisture estimates. Each daily file is approximately 2 Mb, with one year of data equating to approximately 700 Mb. The entire archive from 1983–2024 is approximately 30 Gb.

## Technical Validation

### Comparison against root zone soil moisture datasets

TAMSAT-SM estimates (derived as a weighted sum of the volumetric soil moisture (JULES variable: *smcl*) over the top 1.0 m) were compared to equivalent estimates (over the top 1.0 m) from five other operational datasets used widely in drought monitoring and research^29,30^. These datasets, summarised in Table 4, comprised of three products from NASA’s Land Data Assimilation System (the Global Land Data Assimilation System (GLDAS^31^) Version 2.1 (hereinafter GLDAS-2.1), the GLDAS Version 2.2 (hereinafter GLDAS-2.2) and the Famine Early Warning System Network (FEWS NET) Land Data Assimilation System (hereinafter FLDAS)), the European Centre for Medium-Range Weather Forecasts (ECMWF)’s Reanalysis v5-Land (hereinafter ERA5-Land) and NASA’s Soil Moisture Active and Passive Level 4 soil moisture product (hereinafter SMAP-L4). Our aim here was not to rank the performance of the RZSM datasets but to contextualise the TAMSAT-SM values. Specifically, we sought to (1) evaluate the similarity between TAMSAT-SM temporal and spatial patterns compared to those given by the other RZSM datasets and (2) determine where the absolute values of TAMSAT-SM fits with respect to these datasets that are derived from diverse methodologies and input data sources, and characterised by varying spatial and temporal resolutions.

**Table 4:** Summary of the root zone soil moisture datasets used in this study.

Name (Source)	Soil layers (cm)	Units	Land surface model	Precipitation forcing	Start date	Temporal and spatial resolution
TAMSAT-SM v2.3.1 (University of Reading)	0–10, 10–35, 35–100, 100–300	kg/m^2^	JULES	TAMSAT v3.1	1^st^ Jan 1983	1 day, 0.25° × 0.25°
GLDAS-2.1 (NASA)	0–10, 10–40, 40–100, 100–200	kg/m^2^	Noah-LSM	GPCP	1^st^ Jan 2000	3 hours, 0.25° × 0.25°
GLDAS-2.2 (NASA)	0–2, 2–100	kg/m^2^	Catchment-LSM	ECMWF-IFS	1^st^ Feb 2003	1 day, 0.25° × 0.25°
FLDAS-Global v001 (NASA)	0–10, 10–40, 40–100, 100–200	m^3^/m^3^	Noah-LSM	CHIRPS v2.0	1^st^ Jan 1982	1 month, 0.1° × 0.1°
SMAP-L4 v7 (NASA)	0–5, 0–100	m^3^/m^3^	Catchment-LSM	GEOS, GPM-IMERG	31^st^ Mar 2015	3 hours, 9 km × 9 km
ERA5-Land (ECMWF)	0–7, 7–28, 28–100, 100–289	m^3^/m^3^	H-TESSEL	ERA5	1^st^ Jan 1950	1 hour, 0.1° × 0.1°

Both GLDAS-2.1 (Data citation 2) and GLDAS-2.2 (Data citation 3) integrate satellite data, ground observations, and model derived meteorological forcings to generate estimates of land surface states and fluxes globally. GLDAS-2.1 uses the Noah LSM which is forced with daily Global Precipitation Climatology Project (GPCP) precipitation estimates, meteorological forcings from the National Oceanic and Atmospheric Administration (NOAA)/Global Data Assimilation System (GDAS) atmospheric analysis fields and the Air Force Weather Agency’s AGRicultural METeorological modeling system (AGRMET) radiation fields to simulate soil moisture using an “open-loop” configuration, whereby the system is run without any assimilation of observational data (i.e. the model’s state evolves based on the model physics). GLDAS-2.2 is similar to GLADS-2.1, except that it uses (1) the Catchment LSM which is forced with meteorological analysis fields from the ECMWF Integrated Forecasting System (ECMWF-IFS) and (2) data assimilation to include Gravity Recovery and Climate Experiment (GRACE) satellite observations to account for information on terrestrial water storage anomalies. Both GLDAS-2.1 and GLDAS-2.2 are produced in two modes; a short-latency product which uses fewer inputs dependent on the timeliness of the forcing data (early production stream) and a delayed product which uses the complete set of inputs as described above (main production stream). The main production stream versions of GLDAS-2.1 and GLDAS-2.2 are used in this study, thus providing the most accurate versions of these products for comparison.

FLDAS (Data citation 4) is a custom instance of NASA’s Land Data Assimilation System and is similar to GLDAS-2.1, except its outputs are designed to assist FEWS NET’s decision support efforts across different global regions^32^. For Africa, the FLDAS’s global configuration (FLDAS-Global) provides RZSM based on the Noah LSM which is forced with precipitation estimates from the satellite-based Climate Hazards Group InfraRed Precipitation with Station data (CHIRPS) Version 2.0 product and meteorological analysis fields from the Modern-Era Retrospective analysis for Research and Applications Version 2 (MERRA-2) reanalysis. Analogous to TAMSAT-SM, FLDAS provides full water balance variables (including soil moisture, evapotranspiration and runoff). Importantly, the use of CHIRPS ensures that the FLDAS RZSM estimates are consistent with precipitation estimates that have demonstrated skill over Africa^13,16^. Unlike the GLDAS products, FLDAS provides outputs back to 1982, which are important for characterisation of long-term variability and trends in drought. However, the main caveat of FLDAS-Global is that the data are only available at the monthly scale, which may be considered too infrequent for some applications.

ERA5-Land (Data citation 5) is a high-resolution global climate dataset offering hourly data on land-surface variables such as temperature, precipitation, soil moisture at varying depths, and wind^33^. It is based on the ERA5 atmospheric reanalysis^34^ but includes enhancements for land surface properties, such as improved vegetation and soil properties and better representation of surface fluxes, making it particularly useful for studying land-atmosphere interactions, hydrology, and climate impacts at finer scales. The dataset spans from 1950 to the present, providing a valuable resource for long-term changes in land surface components.

SMAP-L4 (Data citation 6) provides global, 3-hourly estimates of surface (0–5 cm) and root zone (0–100 cm) soil moisture since 2015. SMAP-L4 surface and root zone soil moisture estimates are derived from SMAP L-band radiometer brightness temperature (BT) observations from descending and ascending half-orbit satellite passes. BT observations are assimilated, using an ensemble Kalman filter, into NASA’s Catchment LSM, which enables description of the vertical transport of water in the soil column and hence estimates of RZSM. The Catchment LSM is driven by Goddard Earth Observing System (GEOS) observation-based hourly meteorological forcings and GOES precipitation that is scaled to daily estimates from NASA’s Integrated Multi-satellitE Retrievals for the Global Precipitation Measurement (IMERG) product (Late and Final versions). Out of the datasets considered in this study, it is the only product that utilises direct satellite-derived surface soil moisture observations on a contemporaneous basis.

To allow for a fair comparison between the datasets, the soil moisture content of the top 1.0 m (the depth which approximates the root zone) was computed for all datasets. Where necessary, this involved first converting the soil layers for each product (see Table 4) from water content (kg m^−2^) to volumetric soil moisture (m^3^ m^−3^), and then computing the weighted sum of the corresponding layers taking into account the depth of each layer to give the total volumetric soil moisture content for the top 1.0 m. For computing the TAMSAT RZSM, the soil moisture content of the top three soil layers (0–10 cm, 10–35 cm and 35–100 cm) were used.

**Fig. 2:**
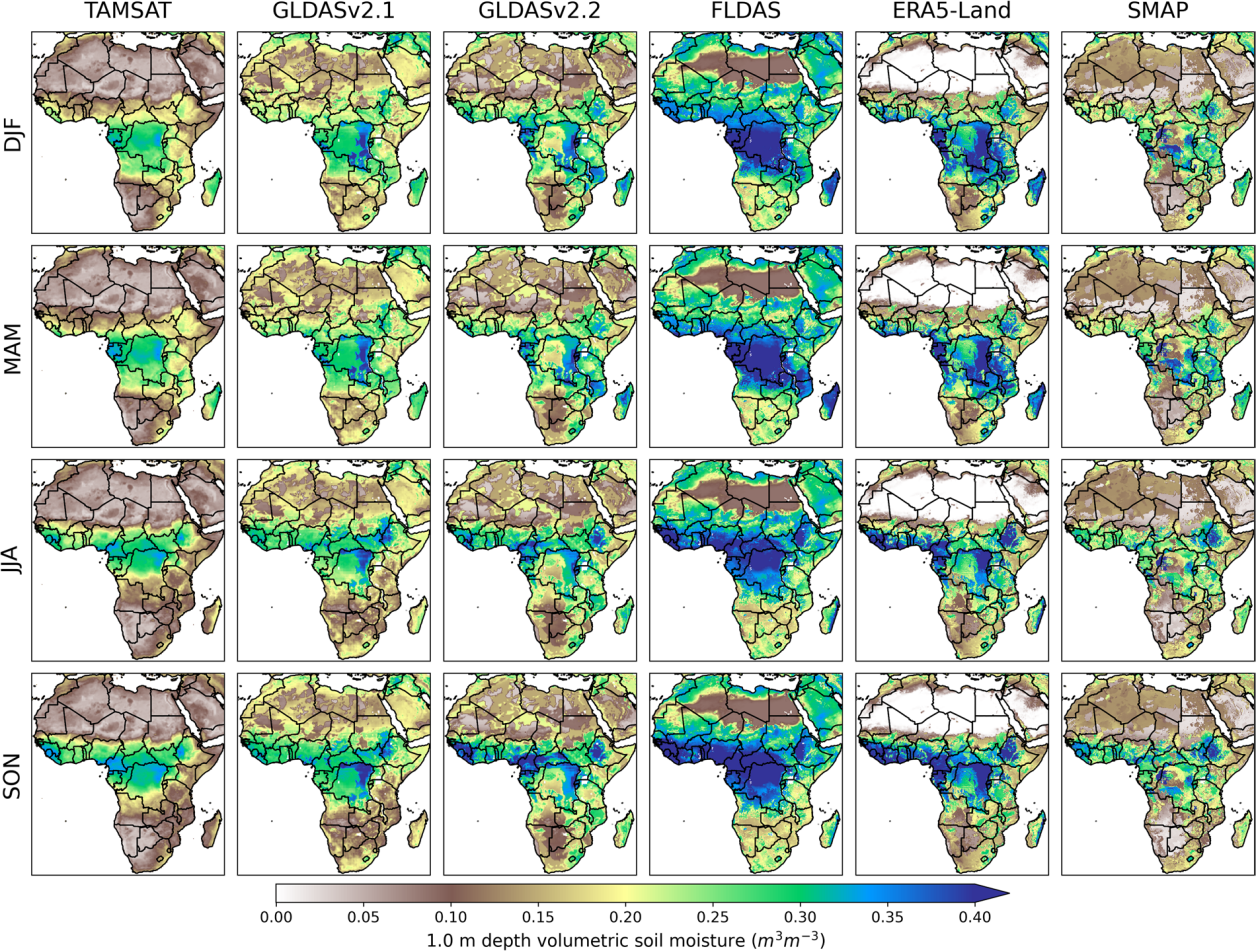
All-Africa seasonal mean (December-January-February (DJF), March-April-May (MAM), June-July-August (JJA) and September-October-November (SON)) RZSM climatologies (derived from 2015–2023) for TAMSAT-SM and the comparison datasets.

Overall, the climatological spatial patterns (see Fig. 2) given by TAMSAT-SM are largely consistent with the other datasets, both in terms of the regions of highest soil moisture values (i.e. south-western region of West Africa, western equatorial Africa, north-east of the Democratic Republic of Congo and the Ethiopian Highlands) and the changes between each season. However, the large variations in the magnitude of RZSM across the products is striking – this is for both tropical regions (e.g. Congo Basin), but also over the semi-arid/arid regions (e.g. Sahel). FLDAS is typically wetter than the other products, while TAMSAT-SM is the generally the driest of the products but is consistent with SMAP in terms of approximate magnitude. This similarity is expected, as SMAP data have been incorporated into the derivation of TAMSAT-SM estimates. SMAP exhibits the greatest spatial variations out of the six datasets – this may be due to the use of direct satellite observations which may capture spatial variations in surface soil moisture better than the other products that are principally model-driven, and possibly missing data.

**Fig. 3:**
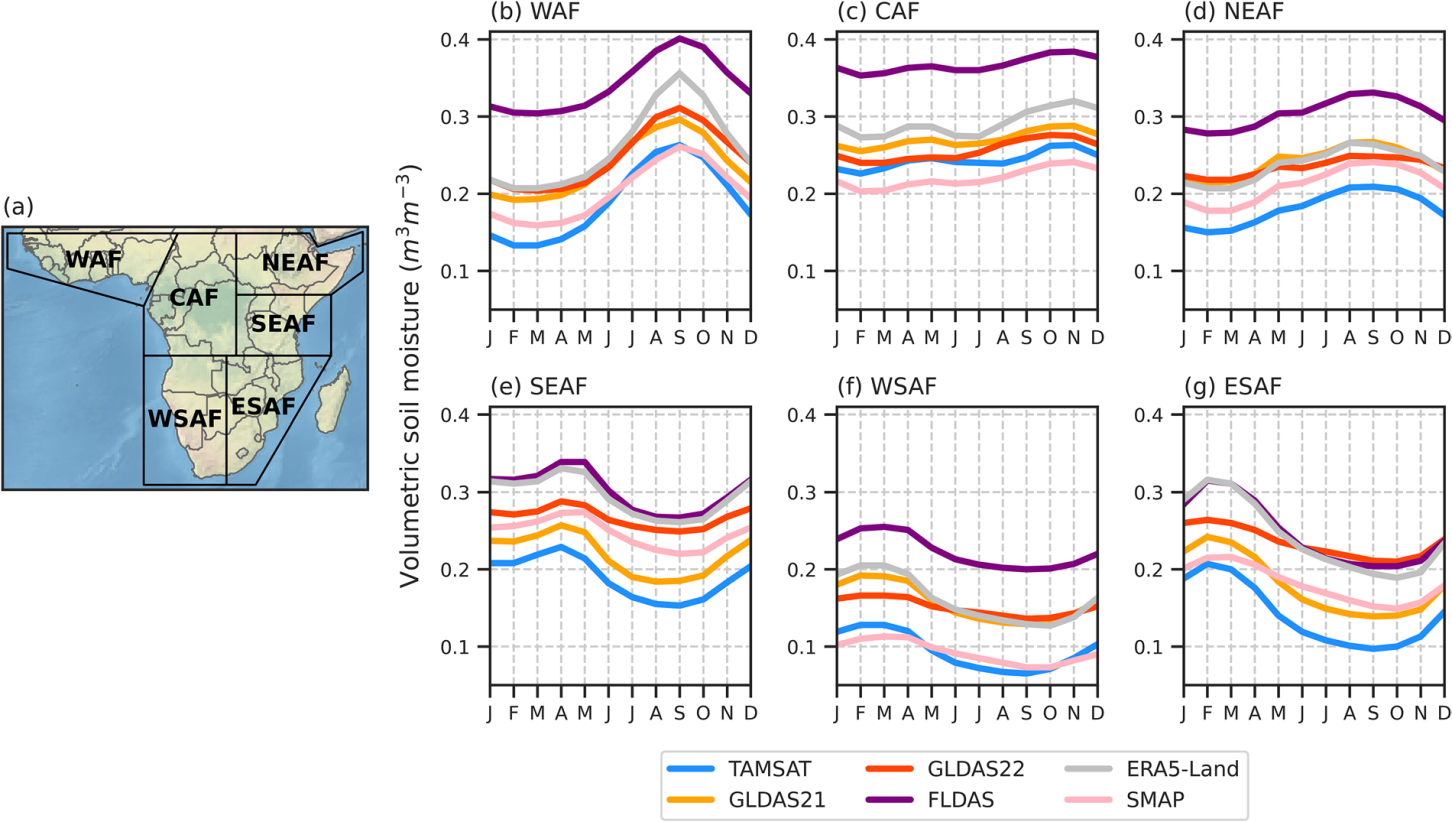
(**a**) IPCC sub-Saharan continental Africa regions (WAF = West Africa, CAF = Central Africa, NEAF = North Eastern Africa, SEAF = South Eastern Africa, WSAF = West Southern Africa, ESAF = East Southern Africa) and the annual cycle (derived from 2015–2023) in monthly mean RZSM for TAMSAT-SM and the comparison datasets for (**b**) WAF, (**c**) CAF, (**d**) NEAF, (**e**) SEAF, (**f**) WSAF and (**g**) ESAF.

The annual cycle in RZSM for each dataset for each of the six sub-Saharan continental regions of Africa used in the Intergovernmental Panel on Climate Change (IPCC) Sixth Assessment Report are shown in Fig. 3 with annual mean RZSM values provided in Table 5. While TAMSAT-SM is generally drier than the other datasets, the annual cycle in TAMSAT-SM is consistent with the shape of the annual cycle given by the other datasets for each of the regions, demonstrating it can reliably capture seasonal variability in RZSM across sub-Saharan Africa. However, the absolute values of RZSM vary considerably between all datasets, as identified in Fig. 2, with the range in RZSM between the six datasets often greater than the range in the RZSM annual cycle for some regions. For example, over SEAF, the range in RZSM between the datasets averaged across all months is 0.11 m^3^m^−3^, while the range in the annual cycle (averaged across the six datasets) is 0.06 m^3^m^−3^. Overall, TAMSAT-SM aligns most closely in magnitude with the SMAP dataset, except in the SEAF and ESAF regions. When comparing TAMSAT-SM to SMAP, it is important to note that there is no state assimilation of SMAP satellite retrievals by TAMSAT-SM, with SMAP only being used to tune JULES’s soil hydraulic properties (described in the Methods section).

**Table 5:** Annual mean RZSM (units: m^3^m^−3^) for each dataset for each Africa region over the 2015–2023 period.

Dataset	WAF	CAF	NEAF	SEAF	WSAF	ESAF
TAMSAT-SM	0.19	0.24	0.18	0.19	0.09	0.14
GLDAS-2.1	0.23	0.27	0.24	0.22	0.16	0.18
GLDAS-2.2	0.25	0.26	0.23	0.27	0.15	0.23
FLDAS	0.34	0.37	0.30	0.30	0.22	0.25
ERA5-Land	0.26	0.29	0.24	0.30	0.16	0.24
SMAP-L4	0.20	0.22	0.21	0.25	0.09	0.18

**Fig. 4:**
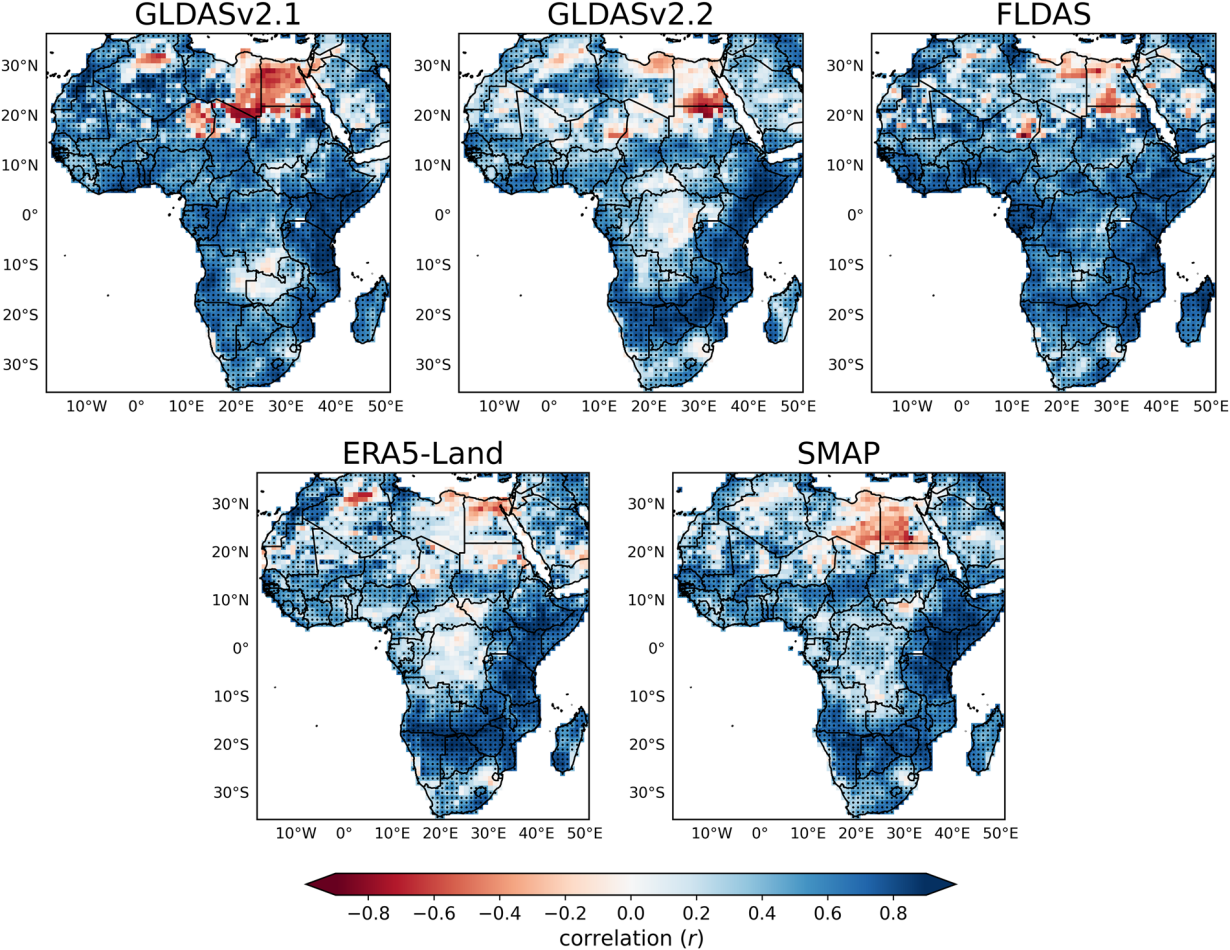
All-Africa spatial correlation between monthly RZSM anomalies of TAMSAT-SM and the comparison datasets between April 2015-December 2023 (anomalies were computed using the monthly climatology derived over this period). Stippling represents statistically significant positive correlations at the 99% confidence level using a two-tailed Pearson correlation test. All data have been regridded to a 1.0° × 1.0° spatial resolution to allow for easier viewing of the stippling.

To assess the ability of TAMSAT-SM to capture temporal variations in RZSM, Fig. 4 provides the Africa-wide grid-scale spatial correlation in monthly RZSM anomalies between TAMSAT-SM and each of the five RZSM datasets between April 2015-December 2023. The use of anomalies enables the assessment of intraseasonal and interannual variability in RZSM caused by anomalous precipitation and/or evapotranspiration, which is most relevant for local assessment of impactful drought. Overall, there is very good agreement between TAMSAT-SM and the other datasets for most of sub-Saharan Africa. East and Southern Africa typically have the highest correlations, exceeding 0.7–0.8 in many areas, although the correlations exceed 0.5 for much of West Africa and the Sahel. There is lower agreement over the Congo Basin, but this is an area where JULES tends to simulate soils close to saturation, and where RZSM is comparatively high all-year round and intraseasonal variability in soil moisture is low (cf. Figure 3c), meaning that variations in RZSM are more difficult to detect. In addition, dense tropical forest canopy cover restricts the penetration of microwave signals, meaning that little or no reliable SMAP soil moisture retrievals are available, reducing SMAP reliability. Lastly, this region is also where satellite-precipitation estimates disagree most strongly over Africa - this is primarily due to a severe lack of rain gauge information which is typically used to either calibrate or merge with satellite rainfall estimates. This further reduces the skill and consistency of driving precipitation estimates over the Congo Basin^35,36^. Considering the datasets in insolation, despite the large difference in absolute values of RZSM, TAMSAT-SM has the best agreement with FLDAS. The high level of agreement between FLDAS and TAMSAT-SM may stem from the fact that both are driven by satellite-derived rainfall estimates (CHIRPS for FLDAS and TAMSAT for TAMSAT-SM), which utilise similar methodologies based on thermal infrared imagery to identify deep convective storm clouds. Similar correlation values were also found when comparing TAMSAT-SM with the other datasets (excluding SMAP) over the 2003–2023 period (not shown). Poorest agreement (given by low or negative correlation values) is found over parts of northern Africa and reflects limitations of JULES in extremely arid and sparsely vegetated environments, where soil moisture processes are poorly represented. TAMSAT-SM is therefore intended for use over sub-Saharan Africa.

While monthly-scale analyses are useful and enable comparison with FLDAS, the ability to detect short-term (e.g., 5–10 day) variations in RZSM is also valuable, as these capture rapid shifts in root zone water availability that directly influence crop growth and provide early warning of developing agricultural drought. Correlation analyses at pentadal (5-day) and dekadal (10-day) scales between TAMSAT-SM and the comparison datasets (except for FLDAS which is only available at the monthly time-step) show a similar level of skill to that found at the monthly scale (see Supplementary Figures S1, S2 for Africa-wide correlation maps, Supplementary Tables S1–S4 for regionally averaged correlations, and Supplementary Figure S3 for time-series comparison over South Eastern Africa (as an example) showing excellent agreement between TAMSAT-SM and SMAP-L4 at multiple time-scales). These results indicate that the performance of TAMSAT-SM is robust across different temporal scales and reflects the persistence of RZSM anomalies over timescales from multiple days to weeks.

**Fig. 5:**
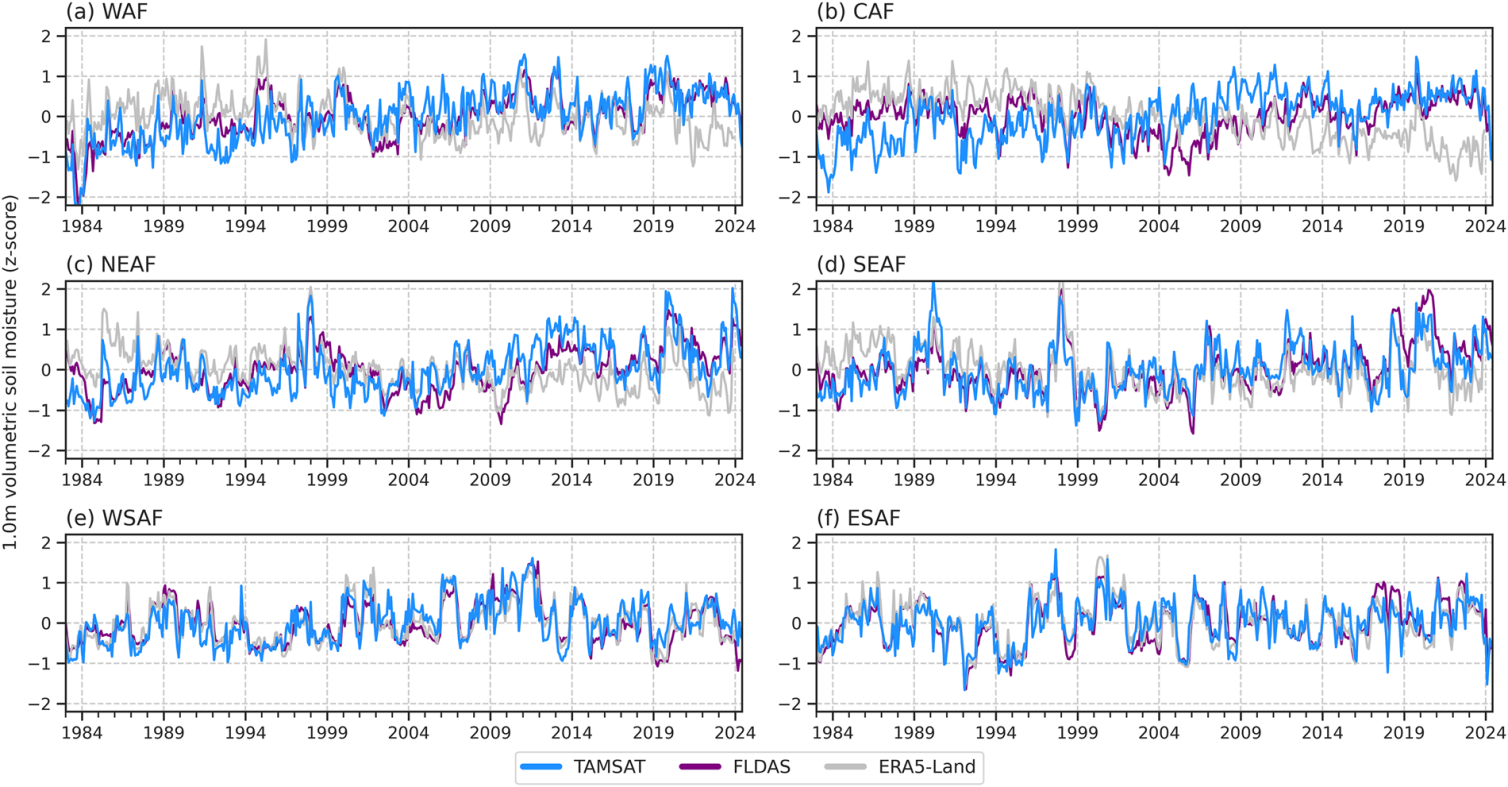
Time-series (1983–2023) of monthly RZSM anomalies, expressed as a z-score, for TAMSAT, FLDAS and ERA5-Land. Z-scores were derived using the 1983–2023 climatology.

**Fig. 6:**
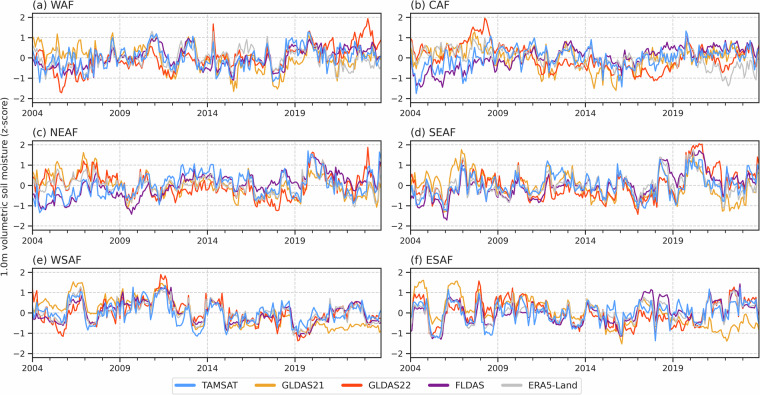
Same as Fig. 5, but for the 2004–2023 period with GLDAS-2.1 and GLDAS-2.2 included. Z-scores were derived using the 2004–2023 climatology.

**Fig. 7:**
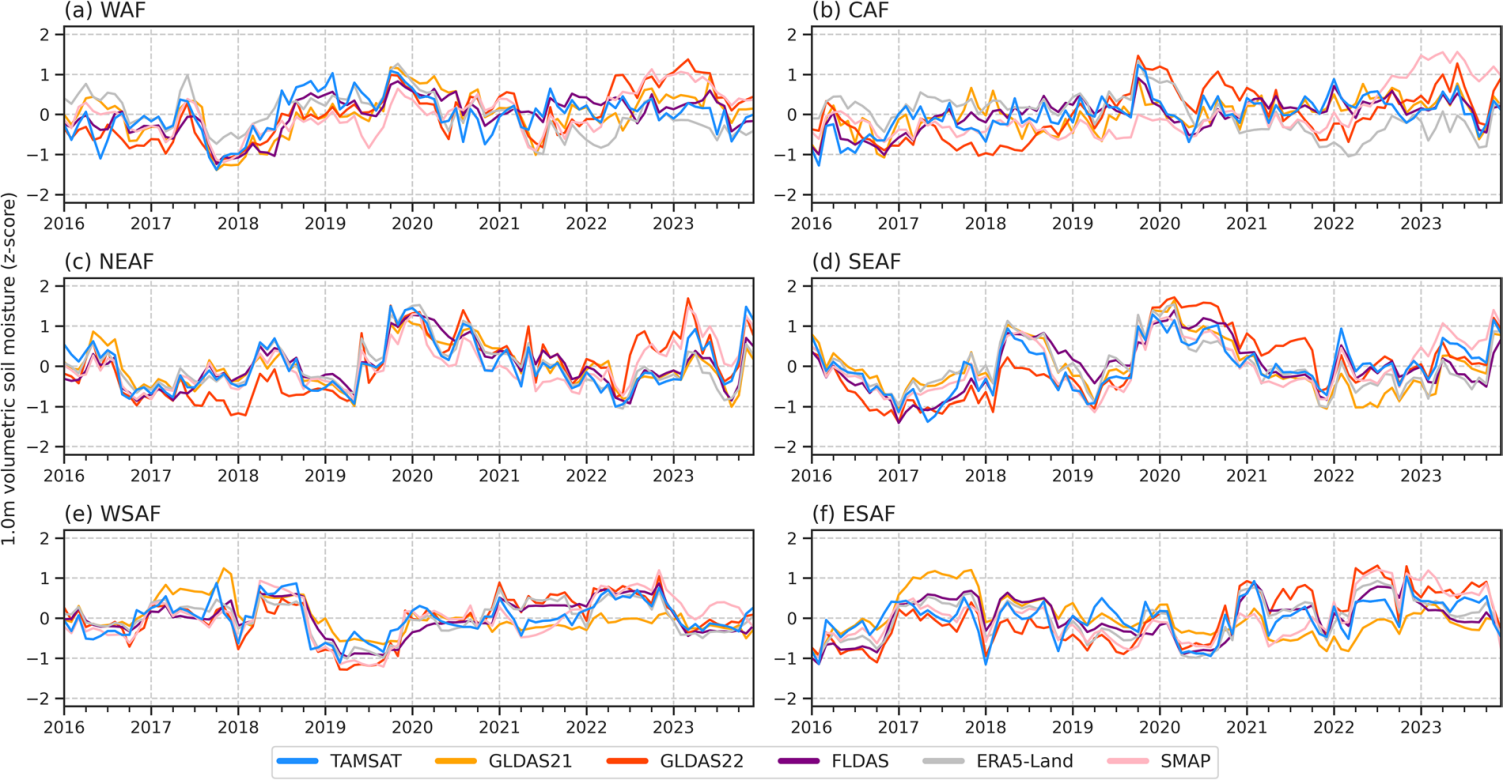
Same as Fig. 5, but for the 2016–2023 period and with SMAP-L4 included. Z-scores were derived using the 2016–2023 climatology.

To assess how consistent TAMSAT-SM is in capturing the temporal variations in RZSM as given by the other RZSM datasets, Figs. 5–7 give the standardised monthly anomalies, expressed as a z-score, in RZSM for 1983–2023, 2004–2023 and 2016–2023 respectively, averaged over the Africa sub-regions. Corresponding correlation values between TAMSAT-SM and the other datasets are given in Table 6. Different periods are used to reflect the different start dates for each of the comparison products and to ensure that the same climatological period is used for each dataset when deriving the z-scores. Given the differences in absolute RZSM values, the use of a z-score, defined below (Eq. 2), measures how many standard deviations a data point is from the mean and allows for a meaningful comparison between the datasets with different climatologies:

2 $$Z=\frac{X-\mu }{\sigma }$$

where $Z$ is the standardised anomaly (z-score), $X$ is the monthly RZSM, $\mu$ is the climatological mean RZSM for that calendar month, and $\sigma$ is the corresponding climatological standard deviation.

**Table 6:** Correlation in the monthly mean RZSM z-scores between TAMSAT-SM and the other RZSM datasets.

Period	Dataset	WAF	CAF	NEAF	SEAF	WSAF	ESAF
Jan 1983-Dec 2023	FLDAS	0.80	0.42	0.71	0.78	0.78	0.81
	ERA5-Land	0.26	−0.30	0.29	0.63	0.84	0.84
Mar 2003-Dec 2023	GLDAS-2.1	0.50	0.33	0.31	0.63	0.67	0.39
	GLDAS-2.2	0.55	0.19	0.36	0.71	0.71	0.66
	FLDAS	0.79	0.68	0.77	0.76	0.78	0.70
	ERA5-Land	0.65	0.18	0.62	0.81	0.85	0.84
Apr 2015-Dec 2023	GLDAS-2.1	0.67	0.63	0.81	0.78	0.55	0.33
	GLDAS-2.2	0.61	0.50	0.74	0.80	0.73	0.67
	FLDAS	0.85	0.83	0.81	0.78	0.75	0.58
	ERA5-Land	0.51	0.03*	0.88	0.78	0.78	0.74
	SMAP-L4	0.52	0.34	0.79	0.89	0.82	0.68

The z-scores were derived for different periods (first column) based on the availability of the comparison datasets to ensure a fair comparison between the z-scores. All positive correlations are statistically significant (using a two-tailed Pearson correlation test) at the p < 0.01 level unless otherwise indicated.

*Not statistically significant (p > 0.01).

Across all three periods, TAMSAT-SM shows very good agreement with the comparison datasets. For example, the correlation between TAMSAT-SM and FLDAS during 1983–2023 for all regions (except CAF) is between 0.70–0.81, with similar correlations during the 2003–2023 and 2016–2023 periods. This suggests that TAMSAT-SM is likely to be skilful throughout its entire record. During 2016–2023 when all comparison datasets are available, TAMSAT-SM again shows consistently high correlation values (mostly > 0.70) for all regions (except CAF) with the comparison datasets, with highest agreement typically over East and Southern Africa (consistent with Fig. 4), indicating TAMSAT-SM can reliably capture intraseasonal and interannual variations in RZSM. It is evident that TAMSAT-SM can also capture significant meteorological events across the continent, such as drought over WAF during 1983–1984 (cf. Figure 5a)^37^ and ESAF during 1992–1993 (cf. Figure 5f)^38^, and wet conditions associated with the 1997–1998 El Niño event over East Africa (NEAF and SEAF; cf. Figure 5c,d)^39^.

Moreover, the comparison between TAMSAT-SM and SMAP (Fig. 7) shows TAMSAT-SM can capture SMAP variations particularly well - with correlations between 0.79–0.89 and 0.68–0.82 over East and Southern Africa respectively - typically better than the correlations between SMAP and the other datasets (see Table 7). This is important as the SMAP-L4 data is, in part, driven by changes in SMAP satellite observations on a contemporaneous basis, providing an independent comparison and a valuable insight into TAMSAT-SM’s ability to capture temporal variations as determined by direct remotely sensed soil moisture observations. While SMAP information is used in the derivation of TAMSAT-SM, as it is used to only calibrate the JULES model in an ‘offline’ mode, interannual variations in TAMSAT-SM are exclusively due to the changes in the meteorological forcing data. This is important as is shows (1) TAMSAT-SM can be reliably used to detect variability in RZSM and (2) that while limited SMAP data (i.e. one year of observations) have been used for calibrating the pedotransfer functions within JULES, the model still yields robust soil moisture information for years outside of the SMAP training dataset.

**Table 7:** Correlation in the monthly mean RZSM z-scores between SMAP-L4 and all other RZSM datasets between April 2015-December 2023.

Dataset	WAF	CAF	NEAF	SEAF	WSAF	ESAF
TAMSAT-SM	0.52	0.34	0.79	0.89	0.82	0.68
GLDAS-2.1	0.76	0.47	0.67	0.81	0.52	0.42
GLDAS-2.2	0.84	0.53	0.79	0.76	0.76	0.79
FLDAS	0.65	0.41	0.72	0.75	0.79	0.72
ERA5-Land	0.36	−0.19	0.68	0.77	0.69	0.80

All positive correlations are statistically significant (using a two-tailed Pearson correlation test) at the p < 0.01 level.

**Fig. 8:**
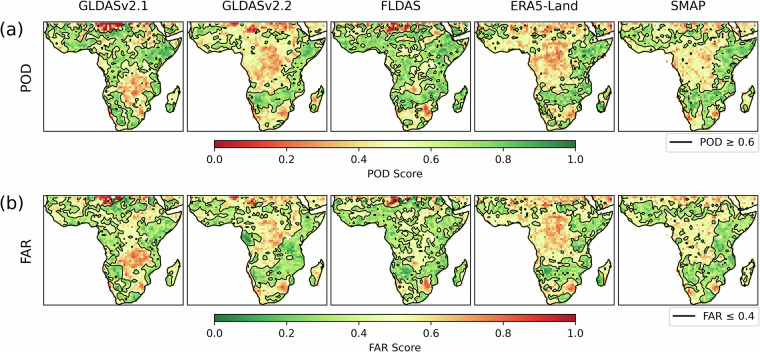
Spatial distribution of (**a**) Probability of Detection (POD) and (**b**) False Alarm Ratio (FAR) calculated from monthly RZSM anomalies, expressed as z-scores, comparing TAMSAT-SM with the comparison datasets for the period April 2015–December 2023. Anomalies were computed relative to the climatology derived over this period, and all data were regridded to 1.0° × 1.0° spatial resolution. Black contour lines indicate regions of good skill (POD ≥ 0.6 and FAR ≤ 0.4).

Since agricultural drought is characterised by periods of low soil moisture, it is important that TAMSAT-SM can accurately detect such conditions. To assess this capability, Fig. 8 shows the spatial distribution in Probability of Detection (POD) and False Alarm Ratio (FAR) of TAMSAT-SM compared against the other RZSM datasets using monthly RZSM anomaly z-scores below the 33rd percentile to define drought – a commonly used threshold used by African National Meteorological and Hydrological Services to identify drought conditions. POD (range 0–1; perfect score 1) is defined as the ratio of correctly detected drought events (hits) to the total number of observed drought events (hits + misses) and reflects TAMSAT-SM’s ability to identify true drought events. FAR (range 0–1; perfect score 0), on the other hand, is defined as the ratio of falsely detected drought events (false alarms) to the total number of detected drought events (hits + false alarms) and indicates TAMSAT-SM’s ability to detect spurious drought events. Together, these two complementary metrics provide an intuitive and balanced measure of performance: a high POD indicates strong sensitivity to true drought conditions, while a low FAR reflects reliability in avoiding false drought events. Figure 8 indicates that the best drought detection skill (given as contours using thresholds of POD ≥ 0.6 and FAR ≤ 0.4) is found across large regions of East and Southern Africa and parts of West Africa, consistent with the spatial patterns of highest correlation skill. Moreover, areas with poorest skill (low POD and high FAR) are mostly found across the Congo Basin – an area that is characterised by saturated soils and low RZSM temporal variability (c.f. Figures 4–7), and which is at least risk of agricultural drought. Overall, this demonstrates that TAMSAT-SM can reliably capture low soil moisture conditions across large regions in sub-Saharan – particularly in places where agricultural drought risk is high.

### Comparison of TAMSAT-SM β against satellite-derived Vegetation Health Index (VHI)

Current agricultural drought monitoring efforts often employ the use of satellite-derived plant health indicators such as those based on vegetation greenness (e.g. NDVI), temperature (e.g. LST) or metrics such as VHI which combines both vegetation and temperature condition indices (VCI and TCI respectively) to assess vegetation health and monitor drought stress. Given the limited availability of *in-situ* data, benchmarking TAMSAT-SM against such products provides a more user-relevant approach to determine (1) the skill of soil moisture datasets (i.e. assessing whether years with low soil moisture correspond to years with poor vegetation health (low NDVI and/or extreme LST) and years with normal/high soil moisture correspond to years with good vegetation health (normal/high NDVI and no extreme LST), and (2) ensures the assessment is based on an independent proxy of agricultural drought. It is important to highlight that different vegetation types (e.g., crops, forests, grasslands) may show different VHI responses even under the same RZSM, meaning the relationship between RZSM and VHI may vary spatially and temporally due to differences in rooting depth, water use, and drought sensitivity. However, aggregating values seasonally helps smooth short-term fluctuations and highlights the dominant patterns in the relationship.

Here, TAMSAT-SM soil moisture availability factor (β) for C4 grasses has been compared to the NOAA Vegetation Health Products (VHP) VHI dataset (Data citation 7), which provides VHI and other vegetation health indicators on a weekly timescale from 1982-present at a 4 km × 4 km spatial resolution^40^. Two gaps in the VHI record (Jan 1985; Sep 1994-Jan 1995) were filled using climatology. To ensure the analysis is relevant to agricultural applications, comparisons have been produced only for regions where staple crops such as maize (a C4 grass type plant) are commonly grown and where there is a recognised and prevalent risk of agricultural drought (i.e. semi-arid regions away from tropical regions as vegetation there is largely unaffected by short-term soil moisture variability). As such, the comparison has been carried out at the regional level for the Sahel, Southern Africa and East Africa (long-rains and short-rains) and considers seasonally averaged values of β and VHI over the respective typical crop growing seasons for these regions (Sahel – June to October; Southern Africa – November to May; East Africa long-rains – March to June; East Africa short-rains – October to January) between 1983–2023.

**Fig. 9:**
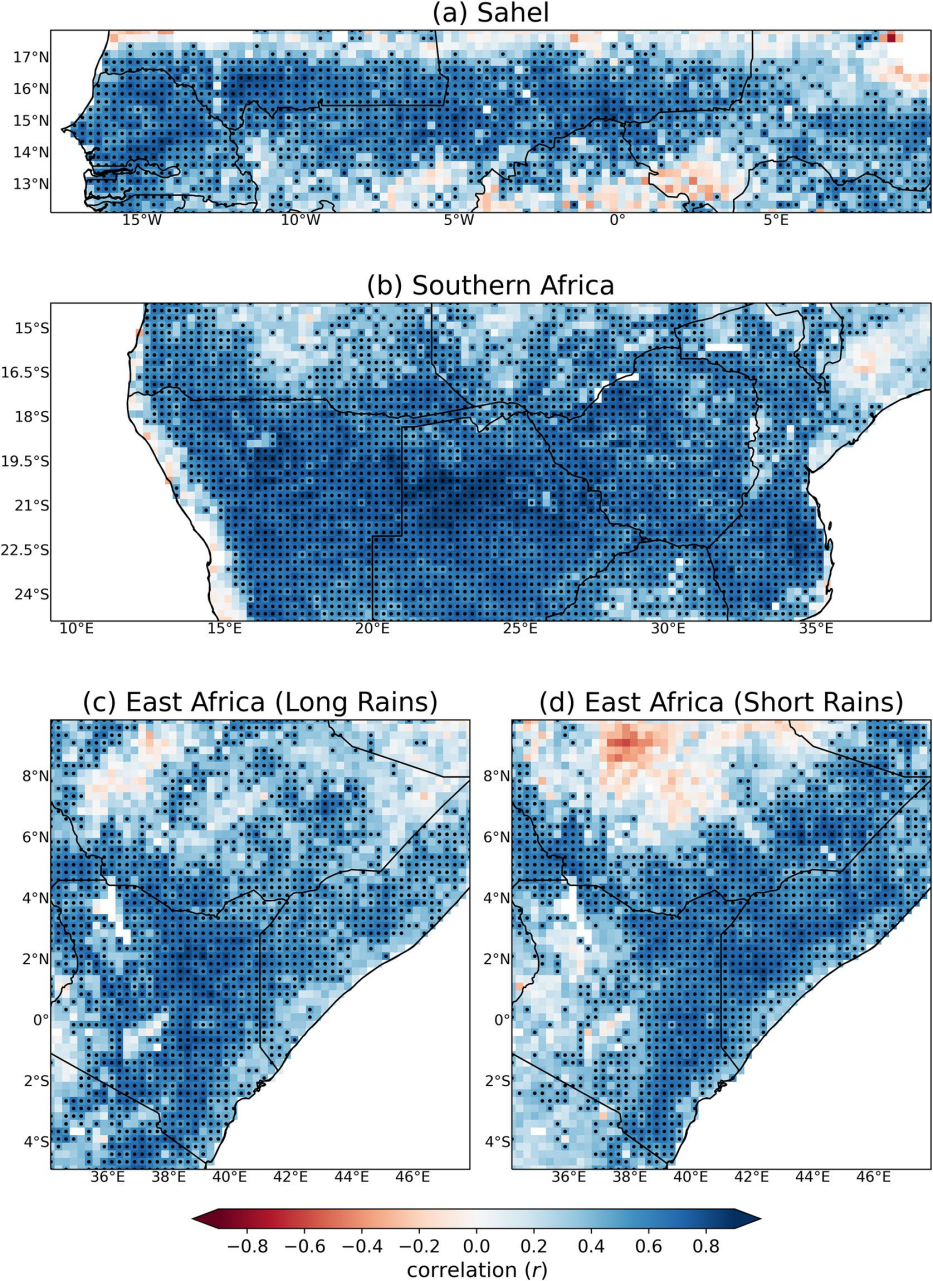
Spatial correlation between seasonal mean TAMSAT-SM (β) and VHI for (**a**) the Sahel, (**b**) Southern Africa, (**c**) East Africa (long rains) and (**d**) East Africa (short rains) over the period 1983–2023. Stippling represents statistically significant positive correlations at the 95% confidence level using a two-tailed Pearson correlation test.

**Fig. 10:**
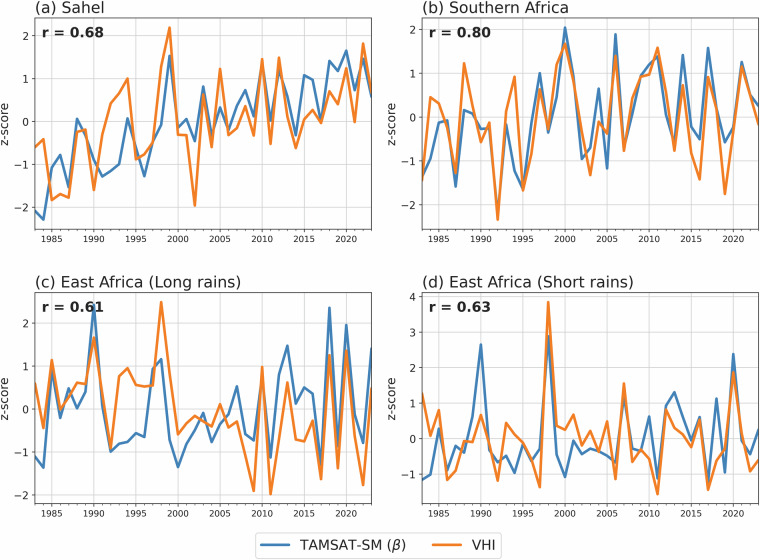
Seasonal mean time-series (z-scores) of TAMSAT-SM (β) and VHI averaged over (**a**) the Sahel, (**b**) Southern Africa, (**c**) East Africa (long rains) and (**d**) East Africa (short rains) between 1983–2023. All stated correlations are statistically significant at the 99% confidence level using a two-tailed Pearson correlation test.

Figure 9 shows the spatial correlation in seasonal β and VHI for each region while Fig. 10 gives the regionally averaged time-series (1983–2023) using all grid squares. For these regions, spatial correlations mostly exceed 0.5, with some areas showing correlations exceeding 0.8, the majority of which are statically significant at the 95% confidence level. When spatially averaged, it is clear there is good agreement between TAMSAT-SM β and VHI for the Sahel and East Africa, with a correlation of 0.68 for the Sahel and 0.61 and 0.63 for East Africa’s long-rains and short-rains respectively (all statistically significant at the 99% confidence level). A notable discrepancy, however, occurs over East Africa (long-rains) in the mid-1990s, where TAMSAT-SM shows a lower z-score than VHI. This may reflect uncertainties in the rainfall forcing (TAMSAT rainfall likely underestimated precipitation during this period (c.f. Figure 3c Maidment *et al*.^41^), with the error propagating into the TAMSAT-SM estimates), but possible issues in VHI during this period, including errors from the gap-filling applied between September 1994 and January 1995, also cannot be ruled out. However, agreement over Southern Africa is markedly better, with a statistically significant correlation of 0.80 (cf. Figure 10b). These comparisons, using an independent dataset (VHI), demonstrate that TAMSAT-SM β can capture interannual variations in vegetation health very well across Africa.

## Usage Notes

The TAMSAT-SM dataset is specifically designed to support agricultural drought monitoring, building upon TAMSAT’s longstanding commitment to assisting African users in climate monitoring and management. This paper has presented a new daily, long-term (1983-delayed present) root zone soil moisture dataset for Africa that provides full column soil moisture and related water balance variables. The TAMSAT-SM soil moisture variables provided are soil moisture availability factor (β) for five PFTs (JULES variable: *fsmc*) and volumetric soil moisture for different soil depths (JULES variable: *smcl*).

In the absence of adequate, Africa-wide *in-situ* data for validation, the new TAMSAT-SM dataset (variable: volumetric soil moisture over the top 1.0 m) has shown to be highly consistent with other commonly used RZSM datasets across Africa, capturing both the climatological spatial and temporal patterns and intraseasonal variations in all regions of Africa except Central Africa – with correlation values typically between 0.7–0.8. Regionally, TAMSAT-SM was most similar to the other RZSM datasets in East and Southern Africa, although good agreement was still evident over West Africa. Central Africa saw the poorest agreement, although visual inspection of RZSM time-series over this region showed less agreement between all datasets indicating this is a region of Africa this is more challenging to monitor. While TAMSAT-SM is drier than the comparison datasets, it is most consistent with remotely sensed soil moisture (SMAP). Moreover, the large range in RZSM values across the datasets highlights the non-trivial nature of RZSM estimation and that the use of the absolute values for any dataset, including TAMSAT-SM, should be done cautiously.

While TAMSAT-SM provides the soil moisture availability factor (β) for all five PFTs, β for C4 grasses, which aligns most closely with staple crop types such as maize but also millet and sorghum (which share similar physiology and soil moisture response characteristics), is recommended as the most suitable indicator of crop water stress for these agricultural systems. Comparison of TAMSAT-SM (variable: β for C4 grasses) with VHI, an independent satellite-derived dataset, between 1983–2023, demonstrated that TAMSAT-SM is largely consistent with vegetation health, where periods of low soil moisture often aligning with poor vegetation health across the Sahel, East Africa and Southern Africa. This consistency supports the use of TAMSAT-SM as a reliable indicator of agricultural drought and vegetation stress across Africa. Moreover, the observed alignment between soil moisture deficits and VHI anomalies highlights the potential of TAMSAT-SM to serve as an early warning indicator for vegetation impacts, particularly when short-term rainfall deficits translate into low RZSM. While regional and land cover differences influence the strength of the relationship between TAMSAT-SM and VHI, the long-term agreement between the datasets underscores the value of integrating soil moisture information alongside vegetation and thermal indices for agricultural drought monitoring and risk assessment.

**Fig. 11:**
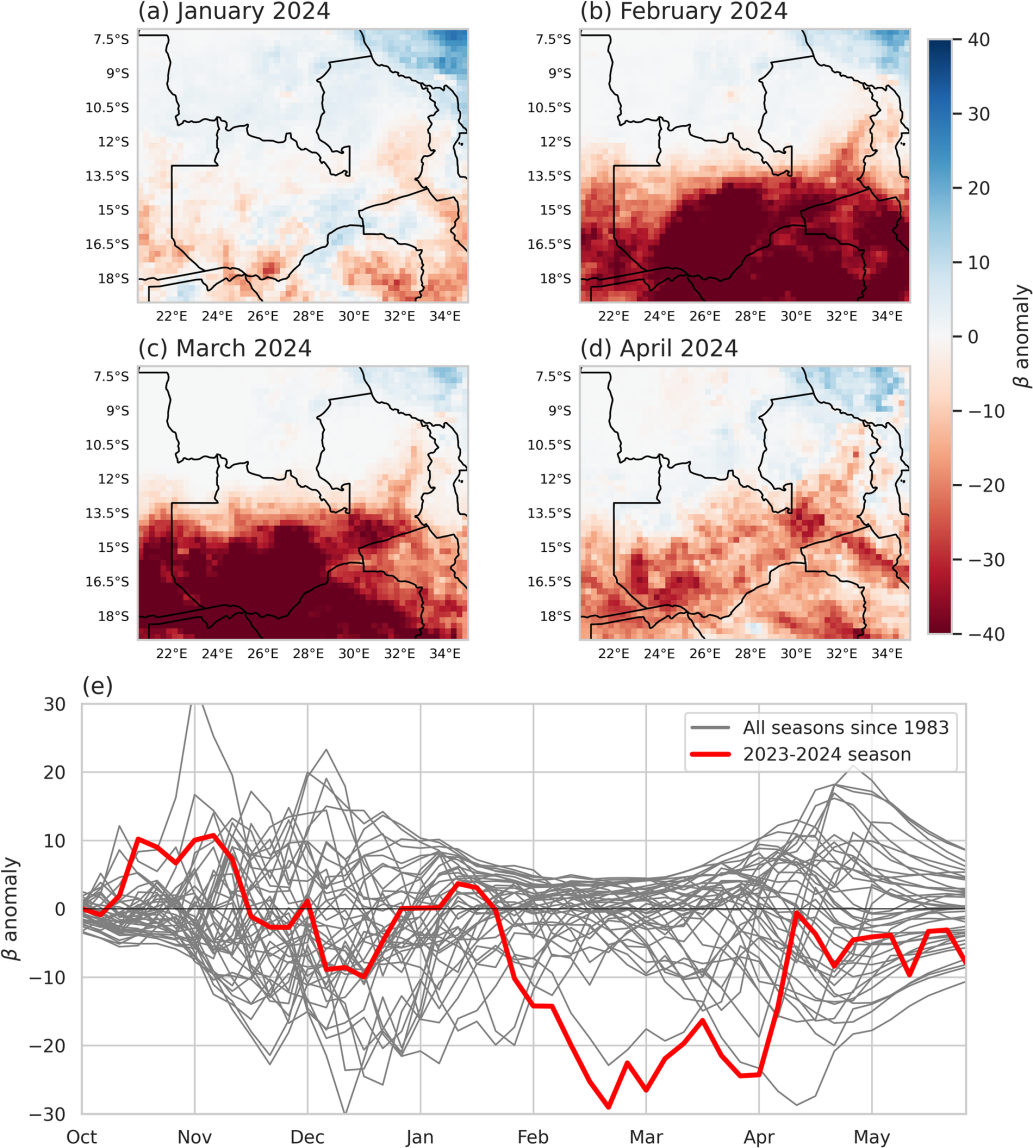
Maps of monthly mean β anomaly (2001–2020 climatology) for (**a**) January, (**b**) February, (**c**) March and (**d**) April 2024 over Zambia and (**e**) pentadal (5-day) mean β anomaly averaged over Zambia for the crop growing season for all years since 1983 (grey lines) and 2023–2024 season (red line).

The most obvious application of the TAMSAT-SM data is its use for NRT monitoring of agricultural drought conditions. A useful demonstration of this is illustrated in Fig. 11, which highlights the utility of TAMSAT-SM soil moisture availability factor (β) for C4 plants (variable; *fmsc*) in capturing the evolution of drought over central Southern Africa in early 2024 that led to widespread crop losses and countries within the region, including Zambia, announcing national emergencies^6^. Figure 11, which shows the (a) spatial and (b) temporal evolution of agricultural drought over Zambia, highlights how the second half of the 2023–2024 season (red line in Fig. 11b) was the driest (in terms of soil moisture) over the 1983–2024 period. The use spatial anomalies (cf. Figure 11a) further show how the spatial extent and severity of the drought evolved during this period. To this end, quick looks of the soil moisture availability factor and soil moisture availability factor anomalies at pentadal (5-day), dekadal (10-day), monthly and seasonal are made available through the TAMSAT website (https://www.tamsat.org.uk) to allow users to easily make qualitative assessments of soil moisture conditions over the period of interest. Given the absolute values of RZSM are inconsistent between datasets, users should be cautious of over-interpreting the meaning of absolute values when using any of the datasets, including TAMSAT-SM and consider relative measures of soil moisture (for both *smcl* and *fsmc*), such as anomalies or z-scores – as demonstrated in this paper. This will ensure comparability with other drought datasets and enable better identification of agricultural drought conditions (i.e. below-average soil moisture during the growing season).

The availability of the TAMSAT data within seven days allows users to make rapid assessments of the presence and severity of agricultural drought while the provision of daily values allows the data to be aggregated over bespoke time-periods. This allows for easy comparison with datasets that are produced at weekly resolutions, for instance, and offers enhanced adaptability for applications other sectors such as hydrology or climate monitoring.

Since the TAMSAT-SM dataset is driven by TAMSAT rainfall data, which has shown temporal consistency over time^14^, it helps ensure temporal stability of the TAMSAT-SM record. This stability is critical for both climate monitoring as well as assessing long-term changes in agricultural drought conditions across Africa. Moreover, the longevity of the TAMSAT-SM record (since 1983) further makes the TAMSAT-SM dataset well suited for drought risk applications where historical information on past drought events is important. A notable example is in developing drought index insurance products for farmers where long time-series are needed to establish reliable benchmarks for pricing of insurance products and determining payouts^42^. Furthermore, consistent skill across multiple evaluation periods demonstrates that the benefits of tuning JULES using a short period of SMAP data (2017) persist throughout the record (as demonstrated in Figs. 5–7 and Supplementary Figure S3).

The compatibility of the TAMSAT-SM data with the TAMSAT-ALERT soil moisture forecasts provides users with utility to combine historical and NRT soil moisture estimates with probabilistic forecasts out to 160 days. This allows proactive decision-making, such as planting date decision support^20,22,23,43^.

TAMSAT-SM does not currently include spatially explicit uncertainty estimates. Deriving uncertainty in RZSM estimates is non-trivial and arises from multiple sources, including rainfall forcing and the representation of land-surface processes within JULES. However, such information is likely to be useful in applications that require formal uncertainty quantification (e.g., risk modelling). As such, developing a framework to provide spatially explicit uncertainty information is a key priority for future development of the dataset.

To conclude, in this paper we have introduced the new TAMSAT daily soil moisture dataset for Africa, providing full-column soil moisture and other water balance variables from 1^st^ January 1983, with updates in NRT (latency < 7 days). Comparative analysis with other RZSM datasets and a satellite-derived vegetation health index demonstrates that TAMSAT-SM performs comparably in skill. This makes it a valuable tool for agricultural drought monitoring and related applications. Its consistency with the TAMSAT rainfall product makes it particularly useful for users already utilising TAMSAT rainfall data who wish to incorporate soil moisture information into their agricultural drought applications.

## Supplementary information

Supplementary Information

## Data Availability

The dataset is available from the CEDA Archive (https://catalogue.ceda.ac.uk/uuid/083f0a37e058495eaef542263198019f/) and the TAMSAT website (https://www.tamsat.org.uk).

## Code availability

We used the Joint UK Land Environment Simulator (JULES), version 5.3 suite u-cs200 for historical estimates (January 1983-Decemeber 2024) and suite u-do123 for operational estimates (January 2025-present). The JULES model code is available from the UK Met Office Science Repository Service (https://code.metoffice.gov.uk/; registration required). A public GitHub release under an open-source licence is in preparation.
