# Supplementary material for: A new, long-term root zone soil moisture dataset for operational agricultural drought monitoring over Africa
Source: Sci Data. 2026 Jan 24;13:260. doi: 10.1038/s41597-026-06585-w (PMC12917200; doi:10.1038/s41597-026-06585-w)
Supplement: Supplementary file 1 — Supplementary Information [file 41597_2026_6585_MOESM1_ESM.docx]

**Supplementary Information**


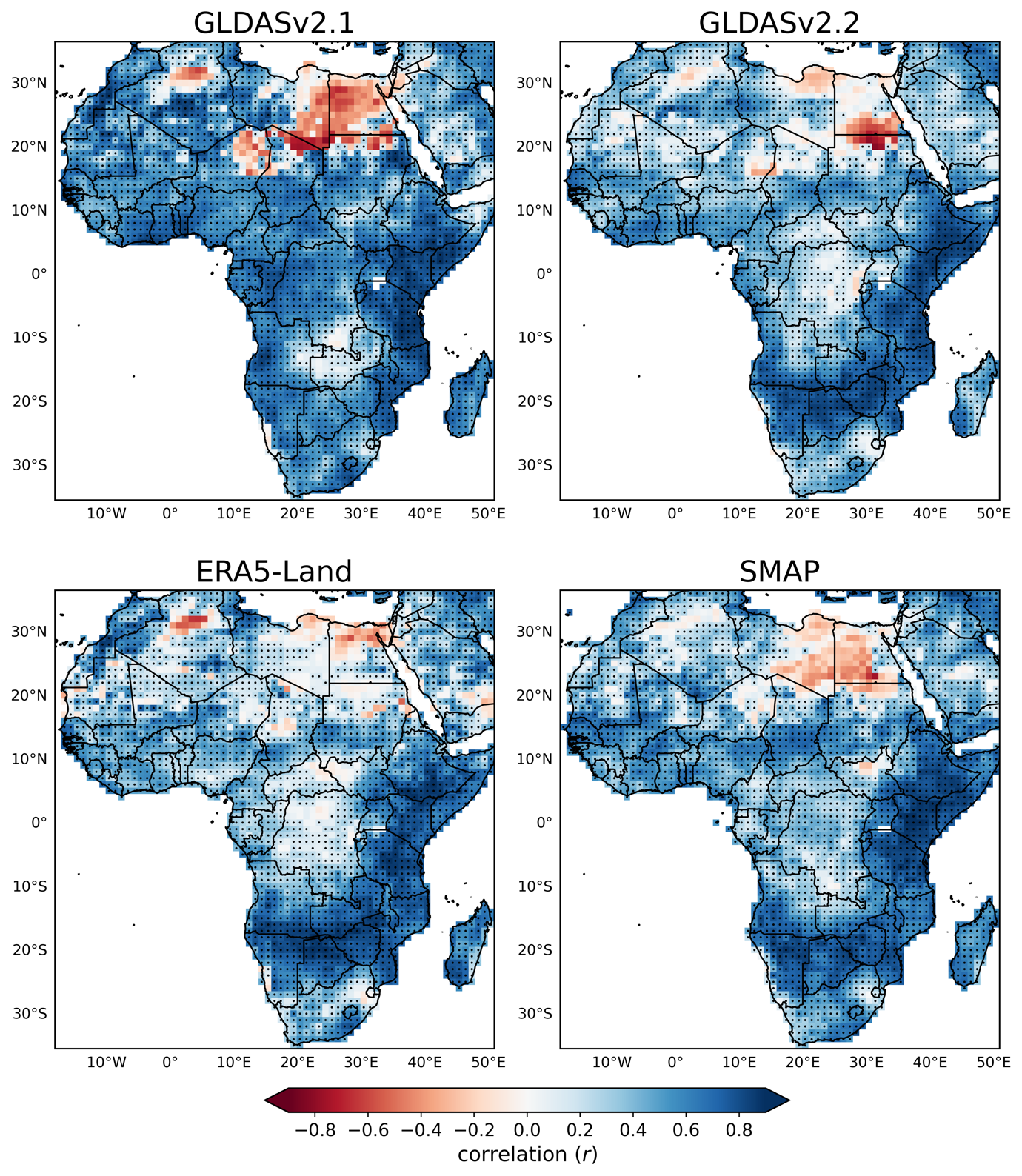


***Figure S1. All-Africa spatial correlation between pentadal RZSM anomalies of TAMSAT-SM and the comparison datasets (GLDADv2.1, GLDASv2.2, ERA5-Land, SMAP-L4) between April 2015-December 2023 (anomalies were computed from the climatology derived over this period).*** ***Stippling represents statistically significant positive correlations at the 99% confidence level using a two-tailed Pearson correlation test. All data have been regridded to a 1.0***$\boldsymbol{^{\circ}}$***x1.0***$\boldsymbol{^{\circ}}$ ***spatial resolution to allow for easier viewing of the stippling.***


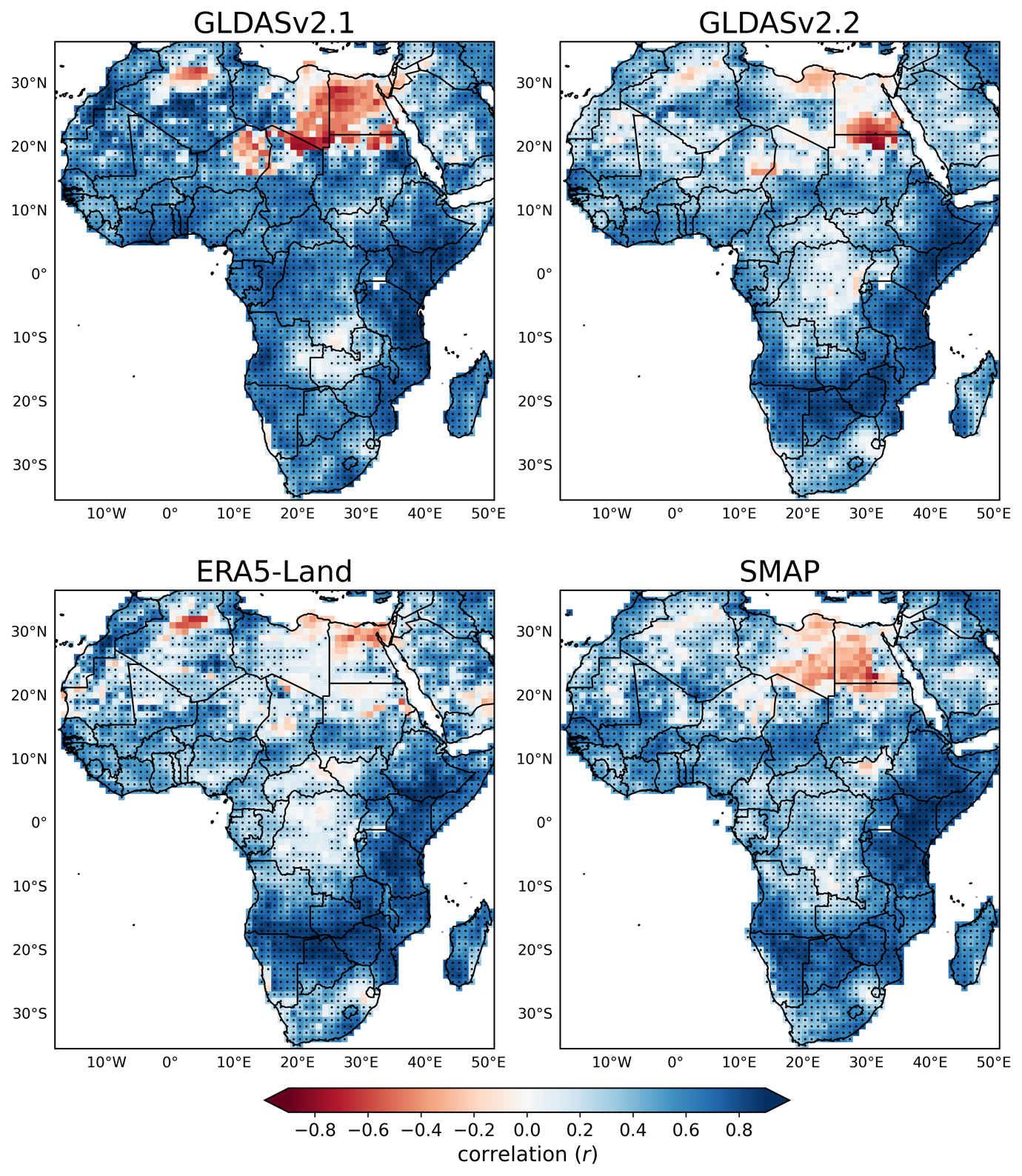


***Figure S2. Same as Figure S1, but for dekadal RZSM values.***

**Table S1. Correlation in the pentadal mean RZSM z-scores between TAMSAT-SM and the other RZSM datasets. The z-scores were derived for different periods (first column) based on the availability of the comparison datasets to ensure fair comparison between the z-scores. All positive correlations are statistically significant (using a two-tailed Pearson correlation test) at the p < 0.01 level.**

| **Period** | **Dataset** | **WAF** | **CAF** | **NEAF** | **SEAF** | **WSAF** | **ESAF** |
| --- | --- | --- | --- | --- | --- | --- | --- |
| Jan 1983-Dec 2023 | ERA5-Land | 0.32 | -0.23 | 0.32 | 0.65 | 0.84 | 0.83 |
| Mar 2003-Dec 2023 | GLDASv2.1 | 0.56 | 0.43 | 0.35 | 0.65 | 0.67 | 0.40 |
|  | GLDASv2.2 | 0.57 | 0.22 | 0.38 | 0.71 | 0.72 | 0.66 |
|  | ERA5-Land | 0.68 | 0.23 | 0.63 | 0.81 | 0.85 | 0.83 |
| Apr 2015-Dec 2023 | GLDASv2.1 | 0.70 | 0.68 | 0.82 | 0.79 | 0.59 | 0.36 |
|  | GLDASv2.2 | 0.62 | 0.54 | 0.73 | 0.8 | 0.76 | 0.67 |
|  | ERA5-Land | 0.55 | 0.13 | 0.87 | 0.79 | 0.78 | 0.74 |
|  | SMAP | 0.54 | 0.36 | 0.80 | 0.89 | 0.82 | 0.69 |

**Table S2. Same as Table S1, but for dekadal RZSM values.**

| **Period** | **Dataset** | **WAF** | **CAF** | **NEAF** | **SEAF** | **WSAF** | **ESAF** |
| --- | --- | --- | --- | --- | --- | --- | --- |
| Jan 1983-Dec 2023 | ERA5-Land | 0.36 | -0.22 | 0.34 | 0.65 | 0.84 | 0.83 |
| Mar 2003-Dec 2023 | GLDASv2.1 | 0.58 | 0.43 | 0.36 | 0.65 | 0.67 | 0.41 |
|  | GLDASv2.2 | 0.58 | 0.23 | 0.38 | 0.70 | 0.72 | 0.67 |
|  | ERA5-Land | 0.69 | 0.25 | 0.64 | 0.81 | 0.85 | 0.83 |
| Apr 2015-Dec 2023 | GLDAS21 | 0.71 | 0.68 | 0.82 | 0.80 | 0.60 | 0.38 |
|  | GLDAS22 | 0.63 | 0.53 | 0.72 | 0.79 | 0.75 | 0.66 |
|  | ERA5-Land | 0.59 | 0.15 | 0.87 | 0.80 | 0.78 | 0.74 |
|  | SMAP | 0.57 | 0.37 | 0.80 | 0.89 | 0.81 | 0.68 |

**Table S3. Correlation in the pentadal mean RZSM z-scores between SMAP-L4 and all other RZSM datasets between April 2015-December 2023. All positive correlations are statistically significant (using a two-tailed Pearson correlation test) at the p < 0.01 level.**

| **Dataset** | **WAF** | **CAF** | **NEAF** | **SEAF** | **WSAF** | **ESAF** |
| --- | --- | --- | --- | --- | --- | --- |
| TAMSAT | 0.54 | 0.36 | 0.80 | 0.89 | 0.82 | 0.69 |
| GLDASv2.1 | 0.78 | 0.50 | 0.68 | 0.81 | 0.54 | 0.41 |
| GLDASv2.2 | 0.85 | 0.55 | 0.80 | 0.76 | 0.78 | 0.79 |
| ERA5-Land | 0.40 | -0.13 | 0.71 | 0.78 | 0.70 | 0.81 |

**Table S4. Same as Table S3, but for dekadal RZSM values.**

| **Dataset** | **WAF** | **CAF** | **NEAF** | **SEAF** | **WSAF** | **ESAF** |
| --- | --- | --- | --- | --- | --- | --- |
| TAMSAT | 0.57 | 0.37 | 0.80 | 0.89 | 0.81 | 0.68 |
| GLDASv2.1 | 0.79 | 0.49 | 0.68 | 0.81 | 0.55 | 0.43 |
| GLDASv2.2 | 0.85 | 0.55 | 0.80 | 0.76 | 0.78 | 0.80 |
| ERA5-Land | 0.44 | -0.10 | 0.71 | 0.79 | 0.70 | 0.81 |

***
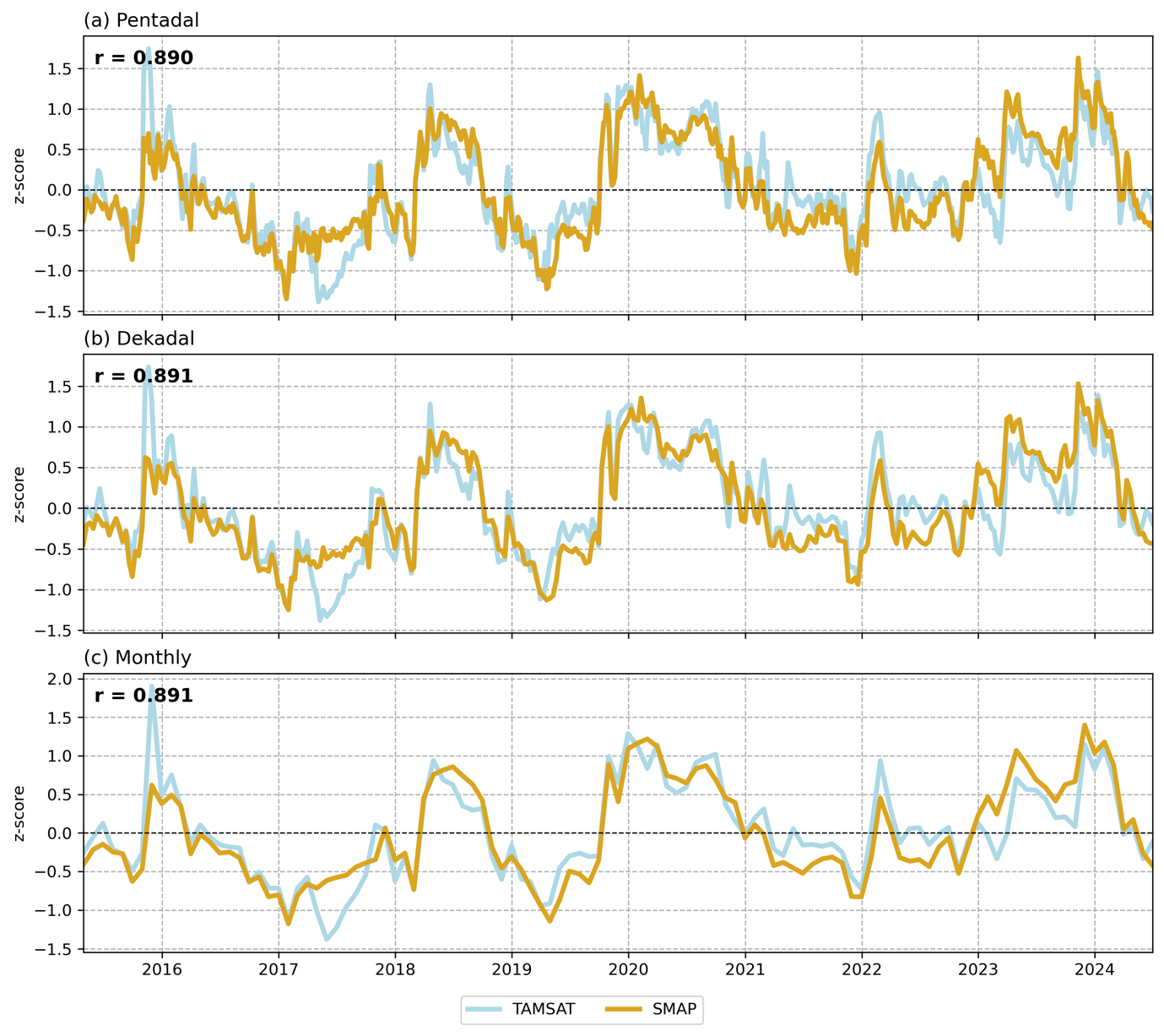
***

***Figure S3. Time-series (April 2015-December 2023) of (a) pentadal, (b) dekadal and (c) monthly RZSM anomalies for South Eastern Africa, expressed as a z-score, for TAMSAT-SM and SMAP-L4. Z-scores were derived using the 2015-2023 climatology.***
